# Climate Justice and One Health Adaptation Policies in Public Health and Disaster Medicine

**DOI:** 10.1002/puh2.70370

**Published:** 2026-09-16

**Authors:** Ioannis Adamopoulos, Aida Vafae Eslahi, Niki Syrou, Konstantina Diamanti, Marilena Katsogiannou, Panagiotis Tsirkas, Maad M. Mijwil, Vesna Tornjanski, Harshit Mishra, Elma Hrustemović, Guma Ali

**Affiliations:** ^1^ School of Social Science Department of Postgraduate Program in MSc Public Health & Policies Hellenic Open University Patra Greece; ^2^ Department of Public Health Policy Sector of Occupational & Environmental Health School of Public Health University of West Attica Athens Greece; ^3^ Medical Microbiology Research Center Qazvin University of Medical Sciences Qazvin Iran; ^4^ Department of Physical Education and Sport Science University of Thessaly, Karies Trikala Greece; ^5^ Department of Early Childhood Education University of Ioannina Ioannina Greece; ^6^ School of Law European University Cyprus Nicosia Cyprus; ^7^ Department of Obstetrics and Gynecology General Hospital of Ioannina “G. Hatzikosta’’ Ioannina Greece; ^8^ College of Administration and Economics Al‐Iraqia University Baghdad Iraq; ^9^ Computer Techniques Engineering Department College of Engineering Technologies Al‐Iraqia Science University Baghdad Iraq; ^10^ Faculty of Organizational Sciences University of Belgrade Belgrade Serbia; ^11^ INTI International University Nilai Malaysia; ^12^ Department of Agricultural Economics College of Agriculture Acharya Narendra Deva University of Agriculture and Technology Ayodhya Uttar Pradesh India; ^13^ Department of Genetics and Bioengineering Faculty of Engineering Natural and Medical Sciences International Burch University, Francuske revolucije bb, Ilidža Sarajevo Bosnia and Herzegovina; ^14^ Department of Food Safety and Environmental Protection Faculty of Veterinary Medicine University of Sarajevo Sarajevo Bosnia and Herzegovina; ^15^ Department of Computer and Information Science Faculty of Technoscience Muni University Arua Uganda

**Keywords:** climate adaptation, climate justice, disaster medicine, health equity, One Health, public health policy

## Abstract

**Background:**

Climate change is intensifying public health risks and placing increasing pressure on disaster medicine systems. These challenges require adaptation strategies that incorporate climate justice principles and the One Health framework.

**Objective:**

This scoping review aimed to examine how climate justice and One Health principles are incorporated into public health and disaster medicine adaptation policies.

**Methods:**

A scoping review was conducted according to the Preferred Reporting Items for Systematic reviews and Meta‐analyses extension for Scoping Reviews (PRISMA‐ScR) guideline using major scientific databases (Scopus, PubMed and Web of Science) and global health organization resources published between 01 January 2005 and 31 October 2025. Critical appraisal of included evidence was assessed using design‑specific tools: the JBI Critical Appraisal Checklist for systematic reviews and the Mixed Methods Appraisal Tool (MMAT) for mixed‑methods studies. Data were synthesized to assess the degree of public health engagement in climate adaptation and the incorporation of climate justice and One Health principles.

**Results:**

Out of a total of 19,835 records, eight eligible studies were included. The eight studies comprised six review articles and two mixed‑methods studies, with geographic coverage, including Pakistan, the United States, Greece and multi‑country or global analyses. Findings reveal persistent inequities in climate‐related health impacts, particularly among marginalized and resource‐limited populations. Health sector participation in national adaptation plans remains limited, with policies often fragmented, mitigation‐oriented or insufficiently aligned with One Health principles. Structural inequalities, weak governance, gender disparities and limited multisector coordination hinder equitable adaptation. Climate stressors, including extreme weather events, air pollution, vector‐borne diseases and environmental degradation, disproportionately affect communities with the lowest adaptive capacity.

**Conclusions:**

There is an urgent need for standardized vulnerability assessment frameworks, stronger multisector collaboration, improved early‐warning systems and enhanced capacity building through international public health institutions.

## 1 Introduction

A serious and expanding hazard to human health is climate change [1]. Developing nations are expected to have the most severe health effects [2]. The most marginalized groups, which have the fewest resources and the ability to adapt, are disproportionately affected by climate change [3]. Therefore, climate justice and health equality must be considered when taking action to address climate change [4]. Climate justice‐based policies that integrate One Health approaches to climate change adaptation should be implemented across all levels without delay [5]. To help authorities develop integrated, climate justice‐based One Health adaptation strategies that address both direct and indirect health consequences as well as ecological implications, the authors propose an emerging agenda [6, 7].

However, high‐income and industrialized states are disproportionately the source of greenhouse gases that contribute to climate change, low‐income states, Indigenous peoples, women, children, elderly people and other marginalized groups are more severely affected in terms of health and livelihoods, despite having contributed the least to the problem [1, 5]. Climate justice accordingly requires adaptation and mitigation policies to take these disparities into account and address distributive, recognition and procedural justice [3, 8]. Recognition justice recognizes the historical and structural causes of vulnerability in particular groups, and it respects their rights, knowledge and involvement in policy processes [4]. Procedural justice ensures that affected groups, particularly the most vulnerable, have the right to meaningful participation in climate and health policy processes. Otherwise, adaptation policies could further exacerbate inequalities, for example, if flood defence systems protect the wealthier parts of the city but not slums [6].

One Health is a transdisciplinary approach which acknowledges the interdependency between human health, domestic and wild animals, plants and the environment, such as ecosystem, soil and water [7]. When integrated within a climate justice perspective, One Health suggests that adaptations must be designed not just for the benefit of human health but must also prioritize animal health and the sustainability of ecosystems, particularly in resource‐poor regions [5]. A climate justice‐informed One Health strategy could be exemplified through investment in veterinary public health and wildlife disease surveillance systems in poorer areas not just to prevent pandemics, but to protect livestock that form the livelihood for pastoralists [7]. Climate justice with One Health essentially questions who will bear the heaviest burdens during periods of environmental breakdown, who will be empowered to restore ecosystems, and what role animals and ecosystems will play in creating an equitable human future.

Adaptation to climate change is a crucial worldwide policy problem where not enough progress has been made [8]. To date, the main goal of policy responses to climate change has been to minimize future climate change by lowering greenhouse gas emissions [9]. This strategy, known as mitigation, offers a solid foundation for analysis as long as emissions are increasing and unanticipated outcomes are possible [10]. In other words, climate change would not be as urgent or catastrophic if emissions could be reduced [11, 12]. Nevertheless, human emissions are increasing.

Climate change is expected to have a significant impact, and it would be extremely dangerous if the average world temperature rose by more than 3–6°C [13]. Most facets of human development in underdeveloped and impoverished nations are at risk from climate change [11]. Serious impacts at 1–2°C of warming are already evident; hence, there is a need to consider complementary adaptation policies as a means of addressing unavoidable climate change [14]. Adaptation is a fundamentally different approach to analysis than mitigation. It requires double‑loop systemic learning, which is a process that goes beyond correcting errors within existing policies (single‑loop) to questioning and transforming the underlying norms, assumptions and governance structures that shape those policies [15, 16, 17]. Systemic improvements in justice did not emerge, despite forum dialogue and adaptive governance leading to successful plans for food pathology improvements [18, 19].

Although previous reviews have focused on adaptation in the public health sphere or justice/One Health in separate pieces; no review has previously attempted to synthetically consolidate the interconnections between all three domains—climate justice, One Health and adaptation policy in the field of disaster medicine—into a single scoping review. Current literature is overwhelmingly focused on only human health, has either ignored or considered animal/ecosystem health secondary and focused on the justice dimension as a matter of mitigation (e.g., fairness of emissions burden), rather than on governance of adaptation policy. In light of this, our review offers three novel contributions: (1) It explicitly charts the inclusion (or omission) of distributive, recognition and procedural justice in the context of health adaptation policy; (2) it thematically quantifies how the omission of animal and environmental health is occurring (a deficit rarely systematically documented); and (3) it offers a framework linking climate stressors, health outcomes, systemic barriers and justice issues, to be incorporated directly into adaptation policy and monitoring. By focusing on adaptation (not mitigation) as the interface between justice and One Health, this research has filled an identified knowledge gap, as acknowledged in recent Lancet and World Health Organization (WHO) reports.

The specific aims of this scoping review are to (1) identify and synthesize the peer‐reviewed and grey literature that explicitly associates adaptation policies with climate justice and the One Health framework in the public health and disaster medicine fields; (2) assess the extent to which human, animal and environmental health are incorporated into climate adaptation policies; (3) identify structural, governance and equity barriers to fair and equitable adaptation; and (4) make future research and policy agenda recommendations’ or ‘propose a future research and policy agenda.

## 2 Methods

### 2.1 Search Strategy

A systematic and structured search strategy was adopted to scan peer‐reviewed articles published between 01 January 2005 and 31 October 2025 that discuss adaptation to climate change, climate justice and public health within the scope of the One Health initiative.

The search was performed on 31 October 2025. The date of publication was searched between 01 January 2005 and 31 October 2025. There was no lower date limitation than 2005 to collect two decades of evidence as defined in the protocol.

This scoping review was conducted following the Preferred Reporting Items for Systematic reviews and Meta‐analyses extension for Scoping Reviews (PRISMA‐ScR) guideline (Table S1) [20]. Searches were conducted across major scientific databases, including Scopus, PubMed and Web of Science, using a predefined search string applied to titles, abstracts and keywords. Additional relevant records were identified through websites and platforms of international organizations, such as WHO, European Centre for Disease Prevention and Control (ECDC), European Public Health Association (EUPHA), International Association of National Public Health Institutes (IANPHI), International Development Law Organization (IDLO), American Society of Tropical Medicine and Hygiene (ASTMH), Health in climate change (HIC) and related organizational repositories.

The search strategy combined terms related to climate change, adaptation, public health, One Health and climate justice using Boolean operators (Table 1).

**TABLE 1:** Syntax for string concatenation, date formatting and conditional logic based on each database.

Database	Syntax	Results
PubMed/MEDLINE	((((((((((“climate change”[Title/Abstract] OR “global warming”[Title/Abstract] OR “climate variability”[Title/Abstract] OR “extreme weather”[Title/Abstract]) AND “climate adaptation”[Title/Abstract]) OR “climate adaptation strategies”[Title/Abstract] OR “climate resilience”[Title/Abstract] OR “disaster risk reduction”[Title/Abstract]) AND “public health”[Title/Abstract]) OR “health outcomes”[Title/Abstract] OR “health equity”[Title/Abstract] OR “health security”[Title/Abstract]) AND “one health”[Title/Abstract]) OR “ecosystem health”[Title/Abstract] OR “human animal environment interface”[Title/Abstract]) AND “climate justice”[Title/Abstract]) OR “environmental justice”[Title/Abstract]) AND (“loattrfree full text”[Filter] AND “medlinestatus medline”[All Fields] AND “english”[Language] AND 2005/01/01:2025/12/31[Date—Publication])) AND ((ffrft[Filter]) AND (medline[Filter]) AND (english[Filter]) AND (2005:2025[pdat]))	872
Scopus	(TITLE‐ABS‐KEY (climate change) OR TITLE‐ABS‐KEY (global warming) OR TITLE‐ABS‐KEY (climate variability) OR TITLE‐ABS‐KEY (extreme weather) AND TITLE‐ABS‐KEY (climate adaptation strategies) OR TITLE‐ABS‐KEY (climate resilience) OR TITLE‐ABS‐KEY (disaster risk reduction) AND TITLE‐ABS‐KEY (public health) OR TITLE‐ABS‐KEY (health outcomes) OR TITLE‐ABS‐KEY (health equity) OR TITLE‐ABS‐KEY (health security) AND TITLE‐ABS‐KEY (One Health) OR TITLE‐ABS‐KEY (human animal environment interface) OR TITLE‐ABS‐KEY (human animal environment interaction)) AND PUBYEAR > 2004 AND PUBYEAR < 2026 AND (LIMIT‐TO (LANGUAGE, “English”))	708
Web of Science	(((((((((((((((TS = climate change) AND (TS = Topic)) OR ((TS = global warming) AND (TS = Topic))) OR ((TS = climate variability) AND (TS = Topic))) OR ((((TS = extreme weather) AND (TS = Topic)) AND (TS = environmental change)) AND (TS = Topic))) OR ((TS = climate adaptation) AND (TS = Topic))) OR ((TS = Climate adaptation strategies) AND (TS = Topic))) OR ((((TS = climate resilience) AND (TS = Topic)) AND (TS = disaster risk reduction)) AND (TS = Topic))) OR ((TS = public health) AND (TS = Topic))) OR ((TS = health outcomes) AND (TS = Topic))) OR ((((TS = health equity) AND (TS = Topic)) AND (TS = health security)) AND (TS = Topic))) OR ((TS = One Health) AND (TS = Topic))) OR ((((TS = ecosystem health) AND (TS = Topic)) AND (TS = human‐animal‐environment interface)) AND (TS = Topic))) OR ((TS = climate justice) AND (TS = Topic))) OR ((((TS = environmental justice) AND (TS = Topic)) AND (TS = English)) AND (TS = Languages))) and Open Access and English (Languages) and Open Access	17,655

Search terms included ‘climate change’, ‘global warming’, ‘climate variability’, ‘extreme weather’, ‘environmental change’, ‘climate adaptation’, ‘adaptation strategies’, ‘resilience’, ‘disaster risk reduction’, ‘public health’, ‘health outcomes’, ‘health equity’, ‘health security’, ‘One Health’, ‘ecosystem health’, ‘human‐animal‐environment interface’, ‘environmental justice’ and ‘climate justice’.

### 2.2 Inclusion and Exclusion Criteria

The review focused exclusively on studies examining health impacts of climate change adaptation within public health systems with specific emphasis on adaptation responses incorporating climate justice or One Health principles. Eligible studies met all of the following criteria: (1) included primary research studies (quantitative, qualitative and mixed methods) and review articles (systematic or narrative reviews) relevant to the topic; (2) peer‐reviewed research or official reports/policy documents from recognized health or environmental organizations (WHO, ECDC, EUPHA, IANPHI, IDLO, ASTMH and HIC); (3) focusing on individuals or population‐level health outcomes; (4) explicit relevance to climate adaptation policies, strategies or interventions; and (5) available in full text and published in English.

For grey literature documents (official reports and policy statements from organizational repositories), formal peer review was not applicable. Instead, these documents were included on the basis of (1) organizational credibility and recognized expertise in climate, health or policy domains; (2) clear authorship and publication date; (3) documented evidence synthesis or consultation methodology; and (4) endorsement as an official organizational output.

Studies were excluded if they (1) focused on topics outside the scope of climate change adaptation, public health or One Health; (2) were editorials, commentaries, conference abstracts or opinion pieces without primary data or review content; and (3) were duplicate publications of the same study.

### 2.3 Study Selection

Duplicates were removed prior to eligibility assessment. Two reviewers independently screened titles and abstracts (I.A. and A.V.E.). The two reviewers involved in title and abstract review also screened the full text independently (I.A. and A.V.E.). Conflicts in any phase were resolved by discussion; if discussion did not resolve a conflict, a third reviewer adjudicated the issue (N.S.). No formal measures of interrater agreement (i.e., Cohen's kappa) were computed, but a record was kept of all screening choices and conflicts. Full texts of potentially relevant articles were retrieved and assessed independently by both reviewers. Discrepancies regarding inclusion or exclusion decisions were resolved through discussion and consensus. The final selection included studies that met the predefined criteria and demonstrated adequate methodological quality for inclusion.

All retrieved bibliographic data were extracted into a reference file and used to visualize consensus networks and thematic clusters related to climate‐related impacts on public health.

**FIGURE 1:**
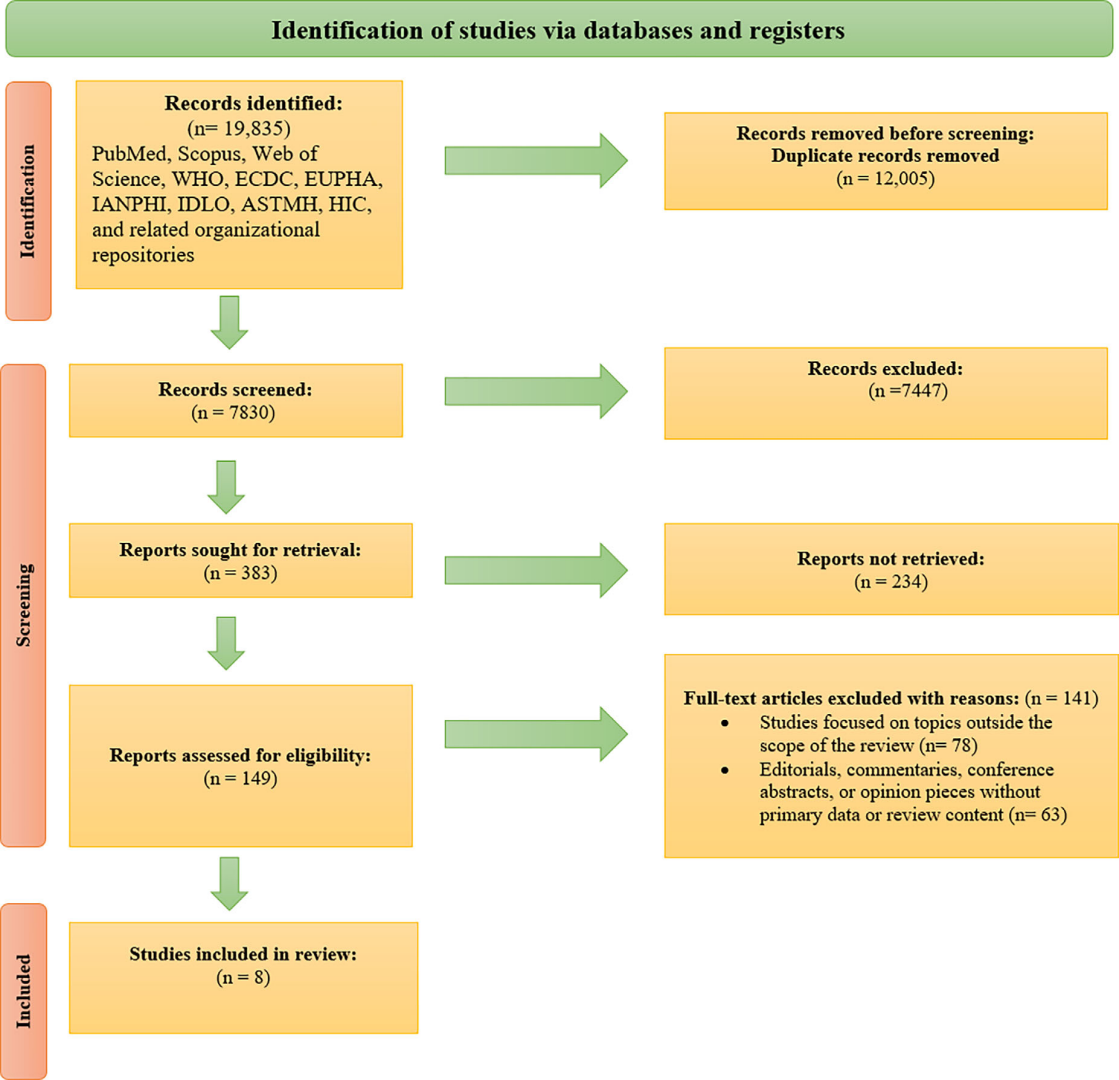
PRISMA flow diagram of the study selection process. ASTMH, American Society of Tropical Medicine and Hygiene; ECDC¸ European Centre for Disease Prevention and Control; EUPHA, European Public Health Association; HIC, Health in climate change; IDLO, International Development Law Organization; IANPHI, International Association of National Public Health Institutes; WHO, World Health Organization.

The full screening and selection process is summarized in PRISMA flow diagram (Figure 1).

### 2.4 Critical Appraisal of Included Evidence

Critical appraisal of included peer‐reviewed evidence was conducted to provide contextual information regarding the methodological strengths and limitations of the included studies and to aid interpretation of findings. Consistent with scoping review methodology, appraisal findings were not used as criteria for study exclusion. Two reviewers (I.A. and A.V.E.) independently performed the appraisal using design‐specific tools. Systematic and review‐based studies were assessed using the JBI Critical Appraisal Checklist for Systematic Reviews and Research Syntheses [21], whereas mixed‐methods studies were evaluated using the Mixed Methods Appraisal Tool (MMAT), version 2018 [22]. For MMAT‐assessed studies, criteria fulfilment was reported descriptively in accordance with MMAT guidance, rather than being interpreted as an overall quality score.

Grey literature documents identified through organizational repositories (e.g., WHO and ECDC) were not subjected to formal peer‐review appraisal tools because these instruments are not designed for policy briefs, technical reports or institutional documents. Instead, these sources underwent a credibility and relevance assessment based on organizational authority, methodological transparency and endorsement status, as described in Section 2.2.

Disagreements between reviewers were resolved through discussion, and when consensus could not be reached, adjudication was undertaken by a third reviewer (N.S.).

### 2.5 Data Synthesis and Statistical Analysis

Eight studies met the final inclusion criteria and were examined using comparative analysis of methodological approaches, statistical techniques and reported outcomes. The review emphasized the rigor, scope and diversity of analytical methods employed across the included studies. Data extraction was performed independently by two reviewers (I.A. and A.V.E.) using a standardized data extraction form. Extracted information was compared, and discrepancies were resolved through discussion.

### 2.6 Conceptual Mapping and Synthesis

**FIGURE 2:**
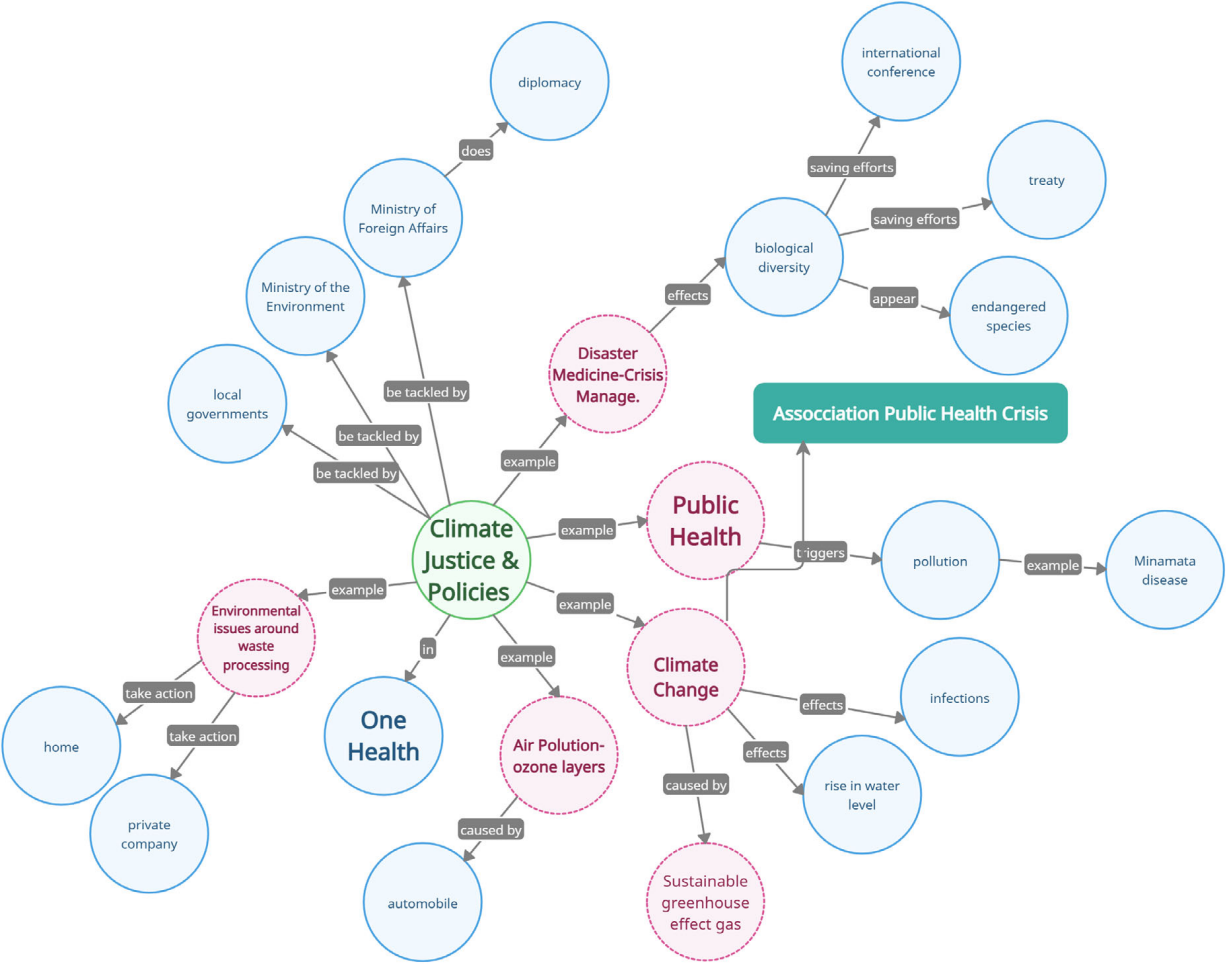
The correlations and associations constitute the key factors of the literature reviews included in this study.

A conceptual map (Figure 2) was constructed to visually integrate key themes (climate justice, One Health, public health adaptation policy, disaster medicine and the interactions there between) that emerged from the included studies. This map was produced using an iterative, qualitative synthesis process. Initially, two authors (I.A. and A.V.E.) individually extracted themes, causal linkages and governance pathways from the eight included studies. Second, extracted items were grouped by themes (climate stresses, health impacts, systemic barriers and justice factors) using affinity diagramming, and third, the relationships between themes were illustrated with directions and links on the basis of both co‐occurrence frequencies in the studies and stated causal relationships between themes (e.g., ‘weak governance limited adaptation capacity and this was seen in disproportionate health impacts’). Finally, the map was tested against the literature by all authors and finalized in consensus. No text‐mining software was used.

The final diagram was produced using Creately (https://app.creately.com), a web‐based diagramming tool to maintain a consistent layout and presentation. Automated text‐mining software or network analysis software was not used to identify themes or relationships. The map is representative of expert, qualitative synthesis of the evidence.

## 3 Results

### 3.1 Study Selection

A total of 19,835 records were initially identified through searches conducted across selected databases. After the removal of 12,005 duplicate records, 7830 unique publications remained for title and abstract screening. During this stage, 7447 records were excluded for lacking relevance to climate change adaptation, public health, climate justice or One Health.

The full texts of 383 studies were sought for retrieval; however, 234 reports could not be retrieved, leaving 149 articles assessed for eligibility. Following detailed review, 141 full‐text articles were excluded for reasons, such as focusing on topics outside the scope of the review (*n* = 78) or consisting of editorials, commentaries, conference abstracts, or opinion pieces without primary data or review content (*n* = 63). Finally, eight studies met all predefined inclusion criteria and were incorporated into the final synthesis.

### 3.2 Characteristics of Included Studies

The eight included studies varied in geographical focus, methodological approach and thematic orientation (Table 2). Together, they addressed health vulnerabilities associated with climate change, the integration of climate justice principles into adaptation frameworks and the role of One Health in strengthening health‐system preparedness. Across studies, climate‐related stressors, such as extreme weather events, air pollution, vector ecology changes and environmental degradation, were consistently linked to disproportionate impacts among marginalized, resource‐limited or socially disadvantaged populations.

**TABLE 2:** A review of the selected studies conducted to examine the primary results and highlight several key findings.

ID	Authors/Year of publication	Study purpose	Methodology	Country/Geographic focus	Outcome/Measures	One Health dimensions captured	Reference
1	Ali et al. (2024)	Identifying and examining the body of research on several sectoral pieces of evidence that affect the environment throughout Pakistan. It seeks to assess the consequences of climate change from a sociocentric perspective	Comprehensive review study	Pakistan	The study found that the research suggested that governmental interference is essential for the sustainable development of the country through strict accountability of resources and regulation implemented in the past for generating state‐of‐the‐art climate policy The findings of the review suggested that GHG emissions cause climate change, which has affected agriculture, livestock and forestry, weather trends and patterns, food, water and energy security and the society of Pakistan	Human, animal, environment (livestock, ecosystems and agriculture)	[1]
2	Abi Deivanayagam et al. (2023)	The Health Policy has three primary aims: to highlight the unequal health impacts of climate change due to racism, xenophobia and discrimination; historical responsibility between countries; and the interplay between climate change, health and discrimination in affected areas globally	A mixed‐method approach to measures, scoping review multi‐method approach to tackle three central aims	Global	This study revealed significant results in many circumstances. Indigenous people, migrants and racially marginalized groups bear an unfair share of the cost of disease and mortality brought on by climate change The findings of this article draw attention to disparities in accountability for climate change and its consequences Outcome measures are responsible for climate change, as well as the estimated mortality and illness risk attributed to climate change per 100,000 people in 2050, geographically	Human only (health inequities, no animal or explicit ecosystem focus)	[4]
3	Odeku (2022)	This study argues that developed industrialized countries are primarily responsible for climate change due to their massive greenhouse gas emissions from their industrial activities. It calls for justice to provide adaptation and mitigation measures through international law instruments and COP agreements	Review study	Global	The results and the relevant findings upon climate injustice are primarily caused by rich developed countries, who contribute to global warming through aggressive industrialization and competition To achieve climate justice, international organizations should implement emission reduction strategies and promote zero‐emission treaties. Fair and equal distribution of climate burden among countries, rich industrialized countries assisting poor countries and sharing the benefits	Human only (climate justice, mitigation and adaptation)	[5]
4	Smith et al. (2022)	Determining and exploring the link between environmental disasters and structural inequities, highlighting how disparities in land use, natural environment and built environment increase vulnerability to these disasters and health disparities	Narrative analytical review	United States	The study indicates a high prevalence of a direct correlation between climate change and increased frequency and intensity of natural disasters in the US, affecting both immediate and long‐term health effects The data‐driven approach critically analyses the role of structural inequalities in climate‐induced health disparities Despite evidence showing, minorities and lower income populations are disproportionately affected	Human, Environment (land use, natural disasters and built environment; no animal dimension)	[6]
5	Campbell‐Lendrum et al. (2021)	The main aims of the study are to illustrate the links between health and climate change and to enumerate the three ‘grand challenges’ for preserving and advancing health in the face of climate change. The examination of the responsibilities, that the health community plays in addressing these issues and promoting change both inside and beyond the health sector, is also included	Review article	Global	The results and the relevant outcome upon the literature show that climate change poses significant health issues, largely affecting health systems A response aimed at reducing carbon emissions, building resilient health systems and implementing public health measures can accelerate the response The scale and scope of the health sector response need to be proportionate to the health threat, requiring political leadership and public support	Human only (health systems, public health measures)	[14]
6	Adamopoulos et al. (2024)	The long‐term impacts of Storm Daniel in Greece, including flooding, landslides, and extreme weather events, have been analyzed in a mixed‐methods review study. Focuses on the effectiveness of mitigation and adaptation strategies and policy evaluating the resilience of urban and rural areas to such events as the environmental and ecosystem effects of the storm and the needs for new guidelines upon climate justice issues	It is a mixed‐method study that utilizes structured modelling techniques, forecasting methods and review includes a variety of strategies and tools used for analysing data	Greece	The findings and the outcomes of this research show that the needs for ecological assessments are crucial for understanding the long‐term effects of climate change on ecosystems Primary to identify potential risks and inform disaster preparedness strategies. It is crucial to measure the promotion of sustainable land use and implement conservation measures for endangered species, we can mitigate future risks Public awareness campaigns and future studies should expand this strategy to better understand the impacts of extreme weather events like Storm Daniel	Human, Animal, Environment (ecosystem effects, endangered species, land use, human health)	[44]
7	Jones (2022)	It aims to explain why climate change is the biggest danger to world health in the twenty‐first century, why immediate action is required to address it and what part health professionals may play in this	Review study	Global	The study highlights that climate action in healthcare can enhance greater resilience to extreme events by reducing emissions of greenhouse gases and promoting climate adaptation. Healthcare professionals play a crucial role in highlighting these threats, advocating for action to protect health and healthcare systems and minimizing environmental impacts in their clinical practice. Healthcare systems and infrastructure must be equipped to manage this unprecedented threat to global health	Human only (healthcare systems, professional roles)	[49]
8	Farley (2022)	Assessing the exploration of the link between resource extraction, prostitution, poverty and climate change, highlighting the negative effects of poverty, choicelessness and the appearance of consent	Review study	Global	The study measures and highlights climate justice and human rights, with resource extraction and poverty being significant issues. Understanding the link between these issues can help implement human rights conventions to reduce harms to survivors, climate refugees and prostituted women. By addressing these issues, we can work towards a more equitable and sustainable future	Human only (human rights, poverty and consent; environment mentioned only as resource extraction context)	[8]

Several studies focused on national adaptation planning and highlighted gaps in multisector collaboration, insufficient integration of ecosystem health and limited participation of the public health sector in climate adaptation governance. Others emphasized the need for community‐driven adaptation strategies, early‐warning systems and risk communication mechanisms that align with both climate justice and One Health principles.

As shown in Table 2, only two of the eight included studies explicitly addressed all three One Health dimensions: human, animal and environment. Four studies focused exclusively on human–health dimensions, whereas two studies included human and environmental dimensions but omitted animal health. This finding reflects a broader gap in the literature: Climate adaptation policies rarely operationalize the full One Health triad, with animal health and ecosystem integrity frequently overlooked.

### 3.3 Synthesis of Findings

#### 3.3.1 Inequities in Climate‐Related Health Impacts

All included studies reported persistent inequities in climate‐related health impacts. Populations most affected by climate stressors included low‐income and marginalized communities, women and girls, informal settlements and rural households, climate‐sensitive occupational groups, and regions with limited adaptive capacity or weak governance. These communities faced compounded risks due to limited access to healthcare, fragile infrastructures and historical social injustices.

#### 3.3.2 Limited Integration of Public Health in Adaptation Policies

Findings revealed a significant gap between national adaptation policies and the degree of public‐health sector engagement. In many countries, adaptation plans remain fragmented, mitigation‐oriented, insufficiently coordinated across sectors or lacking integration of One Health perspectives. National Public Health Institutes (NPHIs) were found to play an inconsistent role, with limited harmonization of risk assessments, surveillance mechanisms or climate‐health preparedness frameworks.

#### 3.3.3 Weak Incorporation of One Health Principles

Although most studies acknowledged the relevance of One Health, its practical implementation within national adaptation strategies was limited. Key deficiencies included little integration of animal and ecosystem health in adaptation strategies, insufficient environmental monitoring to identify early‐warning thresholds, limited cross‐sector data sharing and minimal governance structures to support coordinated One Health action.

Only a subset of reviewed studies showcased models where integrated surveillance or ecological data informed human–health adaptation measures.

#### 3.3.4 Structural and Governance Barriers

Across the studies, structural barriers were identified as major constraints to equitable adaptation: weak governance and fragmented institutions, insufficient multisector collaboration, gender inequalities and patriarchal systems limiting women's participation, inadequate financing and capacity within public health systems, lack of climate justice frameworks within national policies, governance failures amplified climate‐related vulnerabilities and hindered effective implementation of adaptation strategies.

#### 3.3.5 Climate Stressors and Public Health Risks

Included studies highlighted multiple climate‐related stressors affecting health outcomes: heatwaves and extreme weather events, flooding and associated disease outbreaks, air pollution, including dust storms and wildfire smoke, vector‐borne diseases (e.g., malaria, dengue), food and water insecurity and mental health burdens, including climate anxiety.

These stressors were shown to disproportionately affect communities with the lowest adaptive capacity.

### 3.4 Critical Appraisal of Included Evidence

Critical appraisal findings are summarized narratively and presented in Table 3. No study was excluded on the basis of appraisal findings. Across the included review‐based studies, several recurring methodological strengths were identified, including clearly defined review objectives and generally appropriate inclusion criteria. Common methodological limitations included lack of reported independent critical appraisal procedures and limited or unclear reporting of publication bias assessment. In addition, some studies provided insufficient detail regarding search strategies or review processes.

**TABLE 3:** Critical appraisal findings and methodological characteristics of included studies.

Study	Design	Appraisal approach	Main methodological observations
Ali et al. [1]	Systematic review	JBI Critical Appraisal Checklist^a^	Independent critical appraisal and publication bias assessment were not reported
Abi Deivanayagam et al. [4]	Mixed‐methods	MMAT^b^	Integration of qualitative and quantitative components was unclear
Odeku [5]	Review	JBI Critical Appraisal Checklist^a^	Search strategy reporting was incomplete; independent appraisal procedures not reported
Smith et al. [6]	Narrative review	JBI Critical Appraisal Checklist^c^	Publication bias assessment was not reported
Campbell‐Lendrum et al. [14]	Review	JBI Critical Appraisal Checklist^a^	Independent appraisal procedures and search strategy details were unclear
Adamopoulos et al. [44]	Mixed‐methods	MMAT^b^	All MMAT criteria fulfilled
Jones [49]	Review	JBI Critical Appraisal Checklist^a^	Independent appraisal procedures and publication bias assessment were not reported
Farley [8]	Review	JBI Critical Appraisal Checklist^a^	Independent appraisal procedures were not reported; some methodological details were unclear

^a^ JBI Critical Appraisal Checklist for Systematic Reviews and Research Syntheses.

^b^ Mixed Methods Appraisal Tool (MMAT), version 2018. MMAT criteria are presented descriptively and are not interpreted as overall quality scores.

^c^ Narrative reviews were appraised to provide contextual information regarding methodological characteristics and not to generate quantitative quality rankings.

Among the mixed‐methods studies, one study demonstrated minor methodological limitations related to the integration of qualitative and quantitative components, whereas the other fulfilled all assessed MMAT criteria. Overall, the included peer‐reviewed studies demonstrated acceptable methodological rigor, although variability in reporting and methodological approaches was observed across studies.

Grey literature documents identified through organizational repositories (e.g., WHO and ECDC) underwent credibility and relevance screening rather than formal critical appraisal because conventional peer‐review assessment tools are not designed for policy briefs, technical reports and institutional documents.

## 4 Discussion

Climate change is a critical issue that demands immediate attention and coordinated action [23]. Public health policies are being reformulated to prioritize disaster risk prevention, a crucial aspect of climate justice [24]. These policies aim to reduce emissions and increase environmental sustainability, benefiting both health and sustainability [25]. The COVID‑19 pandemic demonstrated that health policy can mobilize rapid, intersectoral action, including enhanced surveillance, risk communication and health‐system surge capacity [26, 27]. Yet, rather than reducing inequities, many countries saw exacerbated disparities in access to care, vaccination coverage and social protection among marginalized groups [28, 29]. Climate change inequity persists [30].

Climate change has significant impacts on health, with increased ill health and suffering being unequally distributed [31]. Social injustices have limited adaptation opportunities for marginalized communities [32]. The current literature lacks an understanding of how climate change impacts social justice and health equity [33]. Climate stressors are commonly evaluated using the exposure–vulnerability–impact model and impacts, but the concepts of justice, equity and health have not been effectively integrated [34]. Climate justice, under the umbrella of social justice, focuses on the distribution of rights, responsibilities and responsive capabilities necessary for health [35].

Climate injustice refers to the systematic, avoidable and unfair distribution of climate change impacts according to social status, including income, race, gender, geography and age [36]. Inequity exists, whereby the groups that least contribute to greenhouse gas emissions consistently experience the most vulnerability to hazards, sensitivity to health impacts and low adaptive capacity [36, 37]. The theoretical relationship with the transdisciplinary framework of One Health lies in their respective distributional logic. One Health, like climate injustice, deals with the unfair distribution of goods and ills of environmental change. It can be argued that, although they deal with distinct domains of existence, their analytical structure is inherently the same.

Climate injustice works at the human–social level. Privilege afforded by money, power and past historical advantages ensure that the greatest share of the burden falls upon those who can afford it the least—Those most exposed to the risks of flood, drought, heatwave and air pollution [36]. One Health takes the distributional logic to the human–social–environment interface. Social marginalization, such as low income and lack of access to adequate food or healthcare, creates a vulnerability to the impacts of environmental degradation (e.g., deforestation, loss of biodiversity and changing land use) and the increased risk of animal disease. For instance, destruction of wetlands for commercial interests may be only a loss of recreation space for the wealthy but may wipe out the only source of food and income for poor fishing communities and also expose them to a higher risk of zoonotic disease transmission due to loss of ecosystem resilience. So it follows that the burden of animal and environmental health burdens can also be unequally distributed according to social status, which is absent in mainstream climate justice thinking.

One Health therefore provides practical ways of tackling climate injustice. Combining the observation of wildlife disease, ecosystem indicators and human–health outcomes with surveillance, the earliest warnings will directly tell policymakers about the impacts on vulnerable populations [38]. Targeted One Health interventions, such as mangrove restoration to protect fishing communities, improving veterinary care for herders or controlling vector‐borne diseases in slums, may decrease disproportionate health effects due to climate change [39]. Ignoring animal and ecological perspectives through which the poor are most exposed to climate impacts (e.g., air‐conditioned houses for the rich vs. vulnerability of animal to changing climate conditions) may lead to maladaptive human‐centred interventions [38]. Hence, a One Health perspective can complement the focus on human‐centred climate adaptation and create a holistic approach to address the unequal burden [39].

The assessment of the impacts of climate change on health must be based on social justice principles, which require models and frameworks for researchers and policymakers [40]. Extreme weather events, starvation, mental disease, respiratory conditions, violence and migration are just a few examples of complex health effects that ripple across social structures [41, 42]. Impacts are more likely to affect marginalized groups, whose public health services are least equipped to handle them [43]. With direct co‐benefits for public health, such as lower air pollution and more active mobility [44], climate change action may also greatly accelerate health equality and justice [45]. There are similarities between climate justice and health equity and justice [46], such as unequal risk distribution and disparities in adaptive ability, with marginalized populations enduring most of the consequences yet bearing the least responsibility, and the key role in monitoring and preventing air pollution is correlated with climate change [47].

According to the results presented in Figure 2, there is a lack of integration between the health sector's engagement in climate adaptation and national climate adaptation policies, which poses a danger to the health outcomes of people worldwide. In regard to research, policy and practice, the need to address the effects of climate change on health is sometimes disregarded in favour of the health sector's climate mitigation and HIC discussions.

Climate justice with global and local governance and government management with the policy adoption of regulations, tools and guidelines affects and controls the global population's public health‐risk factors adoption with adverse impacts. Climate change and extreme weather events are directly correlated with public health and are associated with global crises. Minimizing air pollution plays a central role issues, such as waste, water and wastewater‐reuse processing and management. The strengths of risk assessment tools and inspections should be increased to prevent and control overall crises with holistic scientific fields of disaster medicine, especially with One Health.

Global temperatures are increasing rapidly, prompting urgent actions to limit the increase to 1.5–2°C above preindustrial levels [48, 49]. Health adaptation planning and actions are crucial for addressing the immediate challenges posed by climate change and reducing vulnerability and future exposure [50]. If global warming is limited to 1.5°C, health impacts are projected to be less severe than with an increase of 2°C or more [51]. The current gap in health sector adaptation policies across all levels of governance in South Africa hinders transformational change towards resilience to climate change [52]. Health impacts are a pressing issue within the context of the climate crisis, and health adaptation is urgent and must be addressed in the short term [53], with or without climate mitigation [54]. Climate adaptation at the subnational level is determined by local government actions, with a focus on disaster‐risk reduction in low‐ and middle‐income countries [55]. Every international health organization has the chance to take climate action through the concepts of climate justice, and a ‘just transition’ ought to direct our activities provided by the ASTMH. High‐income nations must quickly decarbonize to lessen the worst consequences of climate change because, historically, they have contributed the most greenhouse gases to the environment [56]. Women rely heavily on informal governance structures for accessing natural resources, reinforcing patriarchal norms and power relations and to ensure equal justice, inclusive climate laws and women's participation in decision‐making, a gender‐transformative approach is needed, with a focus on equality and non‐discrimination [57, 58].

Climate justice involves gender‐inclusive legal, institutional and regulatory processes for climate and biodiversity, empowering women and girls to develop and implement gender‐just climate solutions at all levels, as reported by the IDLO policy brief [59]. The impacts of climate change are causing significant social and environmental damage, necessitating a comprehensive approach to adaptation and mitigation strategies to mitigate potential harm and ensure sustainable development [60]. The policy and policies must balance maintaining societal objectives, allowing compromises and managing potential consequences, ensuring responsible environmental management and a sustainable future in the context of One Health [60, 61].

The IANPHI aims to increase training, competency and capacity among NPHIs in response to heat‐related health concerns [62]. The schools of the environment and public health aim to enhance public health professionals’ understanding and competence [63]. The WHO Europe Working Group HIC promotes communication and collaboration among nations in the WHO European Region [64]. The European Climate and Health Observatory aims to equip public agencies to predict and avoid health risks associated with climate change [65]. Climate‐resilient pathways require management that prioritizes iterative monitoring, and sustainable development objectives reduce disaster risk, promote adaptive management and increase human livelihoods and social and economic well‐being. Enhancing the incorporation of indigenous environmental expertise specific to Copernicus, the Earth observation component of the E.U. Space programme, involves the impacted communities in adaptation planning and promotes candid conversations about political issues and values that influence responses [66].

Assessment frameworks play a crucial role in assessing health risks and vulnerabilities posed by climate change. These frameworks should be standardized and tailored to make it easier for outbreak response professionals to conduct assessments. To ensure social equity, a combined framework should be developed that lists best practices for community engagement and common decision points for each vulnerability assessment.

Additionally, education and training on the application of a standardized framework should be provided to local health departments, as the complexity of assessing vulnerability and health risks can be daunting. Capacity‐building initiatives and training programmes may support implementation of standardized climate‐health assessment frameworks. This will help ensure that vulnerable populations are not overlooked in vulnerability assessments.

Although a substantial body of literature documenting numerous aspects of the negative consequences of climate change has been published, the body of literature focused on addressing ethical obligations concerning adaptation measures has been less well developed. This scoping review analysed the peer‐reviewed literature addressing climate justice and One Health adaptation policies. Gaps in the current literature were identified, limitations of the current review and suggestions for future research were articulated, and implications for action were noted.

Our search revealed a significant shortage of studies directly addressing intersections between disaster medicine, public health, climate justice and One Health adaptation policies. Most retrieved publications were biomedical in focus, with limited attention to systems‐level resilience, ecosystem interactions or integrated adaptation strategies.

Key gaps included are (1) limited incorporation of ecological and animal health dimensions in adaptation research; (2) insufficient analysis of threshold effects, emergent system responses and ecosystem‐level resilience; (3) scarce use of participatory surveillance, community‐based monitoring or long‐term integrated adaptation frameworks; and (4) underrepresentation of policy‐oriented evaluations addressing food security, environmental sanitation and syndromic surveillance systems.

The findings highlight the need for future research to investigate local, regional, national and international One Health system vulnerabilities and resilience within climate adaptation efforts, with improved methodological integration across biological, environmental and social domains.

This study suggests future research on climate‐compatible food and nutrition system policies; examining cultural, social and behavioural dimensions to overcome barriers to policy implementation; assessing community‐driven climate adaptation; evaluating local governments’ capacity to co‐create climate justice evidence and actions; and mapping existing actors for climate justice accountability mechanisms.

Our review also identified that most included studies addressed only human–health dimensions of climate adaptation, with minimal integration of animal and ecosystem health, a gap that underscores the need for more comprehensive One Health operationalization in future research and policy.

Regarding the scope of this research, there were limitations in the jurisdictional confines and the participant pool, limiting the heterogeneity of the sample. This review has several limitations. First, the number of included peer‐reviewed studies was limited, reflecting the relatively small body of literature directly addressing the intersection of climate justice, One Health and public health adaptation. Second, heterogeneity across study designs, geographic settings and thematic focus limited direct comparison between studies. Third, grey literature documents were not subjected to formal peer‐review appraisal tools, although credibility and relevance screening based on organizational authority and methodological transparency was conducted.

## 5 Conclusion

Our findings highlight the fact that inequitable global health systems and human–nature interactions exacerbate the risks climate change poses to health. Across all studies reviewed, the failure of appropriate governance, lack of coherent adaptation strategies and insufficient incorporation of One Health, particularly of the animal and environmental health components were highlighted as the major hindrances to climate adaptation equity, strengthening research capacity is a priority to address these issues.

For many years, researchers and practitioners have focused on heat–health warning systems as an effective method to prevent fatalities and lessen the effects of climate change. Examining the implementation of climate justice principles can enhance disaster resilience and public health.

In addition to increasing awareness of the connections between climate change and public health, particularly health inequities, governments in the environment, health and other sectors must collaborate to address these concerns at all levels, at all levels from local to global.

## Author Contributions

**Ioannis Adamopoulos**: conceptualization, data curation, formal analysis, investigation, methodology, project administration, software, supervision, writing – original draft preparation, writing – review and editing. **Aida Vafae Eslahi**: data curation, supervision, writing – original draft preparation, writing – review and editing. **Niki Syrou**: investigation, methodology, writing – original draft preparation, writing – review and editing. **Konstantina Diamanti**: investigation, writing – original draft preparation, writing – review and editing. **Marilena Katsogiannou**: investigation, writing – original draft preparation, writing – review and editing. **Panagiotis Tsirkas**: investigation, writing – original draft preparation, writing – review and editing. **Maad M. Mijwil**: investigation, writing – original draft preparation, writing – review and editing. **Vesna Tornjanski**: writing – original draft preparation, writing – review and editing. **Harshit Mishra**: writing – original draft preparation, writing – review and editing. **Elma Hrustemović**: writing – original draft preparation, writing – review and editing. **Guma Ali**: writing – original draft preparation, writing – review and editing.

## Funding

The authors have nothing to report.

## Ethics Statement

The authors have nothing to report.

## Conflicts of Interest

The authors declare no conflicts of interest.

## Supporting information




**Supporting File 1**: puh270370‐sup‐0001‐Table1.docx

## Data Availability

All data used in this study are available from the corresponding author upon request. A preliminary version of this work has been deposited as a preprint at https://doi.org/10.20944/preprints202507.0980.v1.
